# Efficacy of external fixation in treating rigid equinus associated with fibro-adipose vascular anomaly

**DOI:** 10.1186/s12891-026-09970-z

**Published:** 2026-05-20

**Authors:** Xiaosong Yang, Ban Lu, Hui Du, Ning Sun, Yan Wang, Ying Li, Wenjing Li, Peng Jiang, Yong Wu

**Affiliations:** 1 Department of Foot and Ankle Surgery, Beijing Jishuitan Hospital, Capital Medical University, Peking University Fourth School of Clinical Medicine, 31 Xinjiekou East Street, Xicheng District, Beijing, 100035 China; 2 Department of Vascular Surgery, Beijing Jishuitan Hospital, Capital Medical University, Peking University Fourth School of Clinical Medicine, 31 Xinjiekou East Street, Xicheng District, Beijing, 100035 China

**Keywords:** Equinus, Fibro-adipose vascular anomaly, Ilizarov, Taylor spatial frame

## Abstract

**Objective:**

To evaluate the efficacy of external fixation in treating rigid equinus deformity associated with fibro-adipose vascular anomaly (FAVA).

**Methods:**

This retrospective study included five patients (four males, one female; mean age 19.4 ± 5.6 years) with FAVA-related rigid equinus who underwent external fixation between January 2021 and August 2023. Three patients were treated with the Ilizarov apparatus and two with the Taylor Spatial Frame. Radiological outcome was measured by the anterior tibiotalar angle (TTA) on weight-bearing lateral radiographs. Functional assessments included the American Orthopaedic Foot & Ankle Society (AOFAS) ankle-hindfoot score, weight-bearing Visual Analog Scale (VAS) for pain, and patient satisfaction using a Likert scale.

**Results:**

The overall follow-up duration was 46.6 ± 13.2 months. The first assessment was performed at a mean of 20.6 ± 13.2 months and the second at a mean of 46.6 ± 13.2 months. At the first assessment, peak ankle dorsiflexion improved significantly from − 43.4 ± 10.4° preoperatively to 4 ± 6.5° (*P* < 0.05); TTA improved from 145.0 ± 18.3° to 106.8 ± 3.0° (*P* < 0.05); and AOFAS score increased from 63.6 ± 4.6 to 75.8 ± 8.0 (*P* < 0.05). Weight-bearing VAS changed from 2 ± 1.2 to 0.8 ± 0.8 (*P* > 0.05). At the second follow-up, AOFAS score was 77.2 ± 12.1 (*P* < 0.05 vs. preoperative) and VAS was 1.4 ± 0.5 (*P* > 0.05 vs. preoperative). The patient satisfaction rate was 80%. Two patients experienced mild recurrence and one patient had severe recurrence.

**Conclusion:**

Gradual correction of rigid equinus deformity secondary to FAVA using external fixation is safe and yields satisfactory short- to mid-term outcomes, providing valuable evidence for the management of this rare condition.

## Introduction

Fibro-Adipose Vascular Anomaly (FAVA) is a rare and complex mesenchymal disorder characterized by fibro-adipose infiltration into the venous structures of muscles and fascia. It primarily presents with localized swelling, tenderness, muscle contractures, and restricted joint mobility. Historically, FAVA has been frequently misidentified as intramuscular cavernous hemangiomas [1–3], with a distinct categorization emerging only in 2014^2^. This high rate of misdiagnosis has often resulted in significant delays in appropriate treatment interventions.

Since the disease commonly involves the calf muscles, many patients with FAVA may eventually develop rigid equinus deformity [4]. The mainstay of treatment for FAVA itself is radical excision [5], while the research on treatment for the associated equinus deformity is sparse and often limited to case reports [1, 2, 6–8]. To our knowledge, there is no literature that specifically addresses the treatment of rigid equinus deformity. The aim of this study is to investigate the efficacy of external fixation for rigid equinus deformity related to FAVA and to explore whether external fixation is a suitable treatment for this condition.

## Methods and patients

We conducted a retrospective analysis of all foot and ankle surgical cases from January 2021 to August 2023. Five patients who met the criteria were included, and their baseline characteristics are listed in Table 1.

**Table 1:** Baseline characteristics, radiologic and functional measurements of patients (*n* = 5)

Case	Gender	AR, y	Side	Follow-up1(mo)	Follow-up2(mo)	Correction Time (w)	External Fixation Time (w)	Preop. TTA	Preop. AOFAS Score	Preop. VAS	Postop. TTA 1	Postop. AOFAS Score 1	Postop. VAS 1	Satisfaction, Likert Scale 1	Postop. AOFAS Score 2	Postop. VAS 2	Satisfaction, Likert Scale 2
1	M	26–30	L	36	62	20	28	145	65	2	104	83	0	SS	71	1	SS
2	M	16–20	R	33	59	6	12	140	56	3	107	69	1	SS	61	2	DS
3	M	16–20	R	16	42	11	17	155	67	0	111	71	1	SS	78	1	SS
4	F	16–20	L	12	38	4	8	118	67	3	104	86	0	VS	93	1	SS
5	M	10–15	L	6	32	12	16	167	63	2	108	70	2	NS	83	2	SS

*Abbreviations*: *1* first follow-up, *2* second follow-up, *AR* age range, *AOFAS* American Orthopaedic Foot & Ankle Society ankle and hindfoot scale, *F* female, *L* left, *M* male, *NS* neither satisfied, *R* right, *SS* somewhat satisfied, *TTA* tibiotalar angle, *VS* very satisfied, *DS* dissatisfied

*P < 0.05

### Inclusion and exclusion criteria

Inclusion criteria: (i) diagnosis of FAVA confirmed by a vascular surgeon; (ii) severe rigid equinus deformity associated with FAVA, as determined through joint consultation between vascular and foot and ankle surgeons; (iii) main treatment with external fixation; (iv) unilateral lesions. Exclusion criteria: (i) incomplete follow-up data; (ii) poor compliance from the patient.

### General information

There were 4 males and 1 female with an average age of 19.4 ± 5.6 years (range, 13–28 years). Three patients were affected on the left side, and 2 on the right side.

Clinical history and preoperative data were extracted from the institutional electronic medical records. The first follow-up assessment was performed in the outpatient clinic, including physical examination, weight-bearing lateral ankle radiography, and completion of functional questionnaires (AOFAS, VAS, and Likert scale). The second follow-up assessment was conducted via telephone interview, during which patients were asked about current symptoms, recurrence of equinus deformity, pain level, and overall satisfaction.

### Surgical procedure

A thorough evaluation was conducted to ascertain the necessity for preliminary vascular surgery. Surgical strategies were developed in close collaboration with vascular surgeons. Case 1 underwent corrective shaping using the Ilizarov apparatus. Case 2 underwent corrective shaping using the Ilizarov apparatus combined with gastrocnemius fasciotomy. Case 3 underwent corrective shaping using the Taylor Spatial Frame. Case 4 underwent corrective shaping using the Taylor Spatial Frame combined with Achilles tendon lengthening and plantar fascia release. Case 5 underwent corrective shaping using the Ilizarov apparatus combined with Achilles tendon lengthening and gastrocnemius fasciotomy.

The surgery was conducted under general or spinal anesthesia with the patient in a supine position. A thigh tourniquet was utilized, and the hip on the involved side was elevated to facilitate internal rotation of the limb. One to two external fixation rings were affixed to the lower leg using either full or half pins. A foot ring, aligned parallel to the plantar surface, was secured with two pins each in the calcaneus and the forefoot, linking the foot to the leg rings. Toes not affected by stiff deformity were left unfixed but underwent daily passive stretching exercises. In instances where a Taylor Spatial Frame was employed, incorporating a complete proximal tibial ring and a foot ring, the setup was accurately marked and calibrated under fluoroscopic guidance. Subsequent adjustments were directed by an internet-based application (Smith & Nephew), which provided a tailored schedule for external fixation adjustments based on entered parameters.

Preoperatively, the extent of FAVA involvement was evaluated using magnetic resonance imaging or ultrasonography. Intraoperatively, incisions were generally avoided within the affected region, and the entry and exit points of the pins were selected to circumvent the FAVA area. When such avoidance was not feasible, meticulous attention was paid to drilling and the depth of half-pin placement. Postoperatively, the rate of distraction was adjusted based on the patient’s pain, neurological status, and vascular response to prevent excessively rapid correction. In cases where concurrent flexion deformity of the knee joint was present, simultaneous correction using an external frame was often required.

### Postoperative care

Postoperatively, external fixator adjustments began in the first week, guided by the absence of pain, performed four times daily at a rate not exceeding one millimeter per day. Patients were instructed on home pin site care and underwent bi-weekly radiographic checks to prevent anterior impingement or dislocation. Adjustments continued until achieving five to ten degrees of ankle dorsiflexion, after which the fixator was maintained for a period to solidify the correction. In the case of significant pin site issues, early fixator removal was considered, followed by stabilization with a cast or brace until the planned timepoint. After fixator removal, patients gradually commenced weight-bearing, using an ankle-restricting walking boot for 4–6 weeks, and utilized a night brace for at least six months to prevent recurrence.

### Outcome assessment

Imaging evaluation was performed using weight-bearing lateral ankle views, assessing the anterior tibiotalar angle (TTA) by measuring the angle between the anatomical axis of the tibia and the axis of the talus, with angles greater than 115° defined as equinus deformity [9].

Functional assessment utilized the American Orthopaedic Foot & Ankle Society (AOFAS) ankle and hindfoot score, with pain scored using the Visual Analog Scale (VAS). Only weight-bearing pain VAS scores were included due to patient pain present only during weight-bearing. Patient satisfaction was measured using five-point Likert scale which described 5 scenarios: very satisfied, somewhat satisfied, neither satisfied nor dissatisfied, somewhat dissatisfied, and very dissatisfied.

### Statistical analysis

Data were analyzed using SPSS software (version 27.0; SPSS, Inc., Chicago, IL, USA). The Shapiro-Wilk test was employed to assess the normality of continuous variables. Continuous normally distributed data were presented as mean ± standard deviation. Given the study’s limited sample size, the Wilcoxon rank-sum test was employed for the statistical analyses. A P-value of less than 0.05 was designated as the threshold for statistical significance.

## Results

The specific data for the five cases are shown in Table 1. This study included two follow-up assessments. The overall follow-up duration was 46.6 ± 13.2 months. The first follow-up assessment was conducted at a mean of 20.6 ± 13.2 months, and the second at a mean of 46.6 ± 13.2 months, with an average external fixation adjustment duration of 10.6 ± 6.2 weeks (range, 4–20 weeks) and a mean total external fixator wear time of 16.2 ± 7.5 weeks (range, 8–28 weeks).

**Fig. 1:**
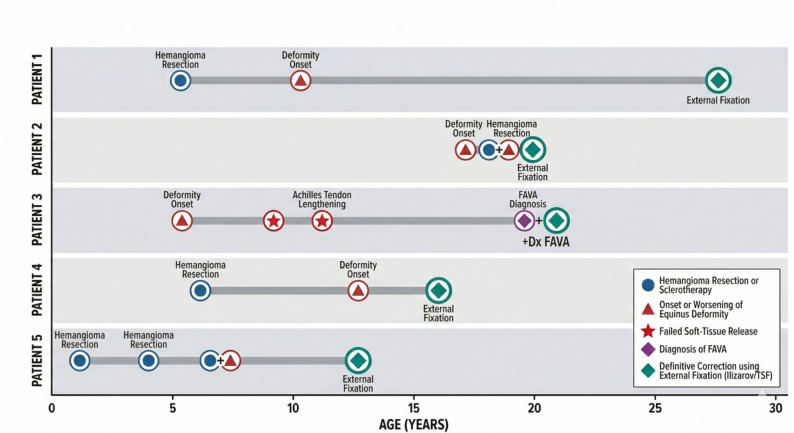
Clinical history timeline of five patients with FAVA-related rigid equinus

Case details were as follows (Fig. 1): Case 1 underwent resection of a left calf hemangioma at age 5 years. Foot deformity developed at age 10 years, and external fixation was performed at age 26–30 years.

Case 2 developed foot deformity at age 18 years. Resection of a right calf hemangioma was performed at age 19 years, after which the equinus deformity worsened. External fixation was performed at age 16–20 years.

Case 3 first presented with equinus deformity at age 5 years. The patient underwent right Achilles tendon lengthening at an outside hospital at age 9 years, with recurrence noted two months postoperatively. A second surgical procedure was performed at age 11 years, but recurrence occurred again three months later. No definite history of hemangioma was initially documented; however, FAVA was subsequently diagnosed at our institution, where the patient received concurrent percutaneous sclerotherapy for a lower extremity arteriovenous malformation. External fixation was performed at age 16–20 years.

Case 4 underwent hemangioma resection at age 6 years. Equinus deformity developed at age 13 years, and external fixation was performed at age 16–20 years.

**Fig. 2:**
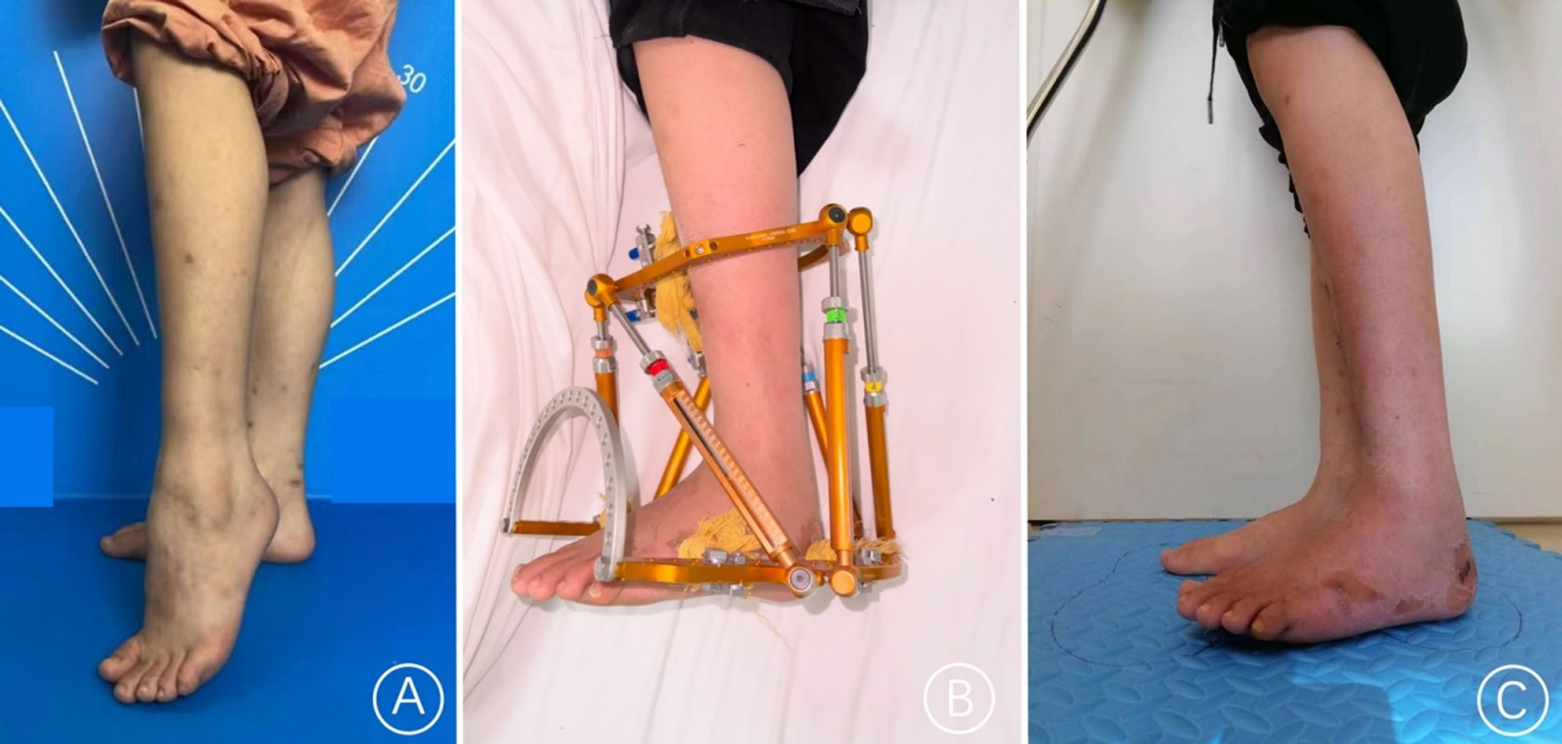
Typical case 5, rigid equinus deformity related to Fibro-Adipose Vascular Anomaly. **A** preoperative appearance of left foot shows significant rigid equinus deformity. **B** postoperative appearance of left foot before fixator removal. **C** appearance of left foot after fixator removal

**Fig. 3:**
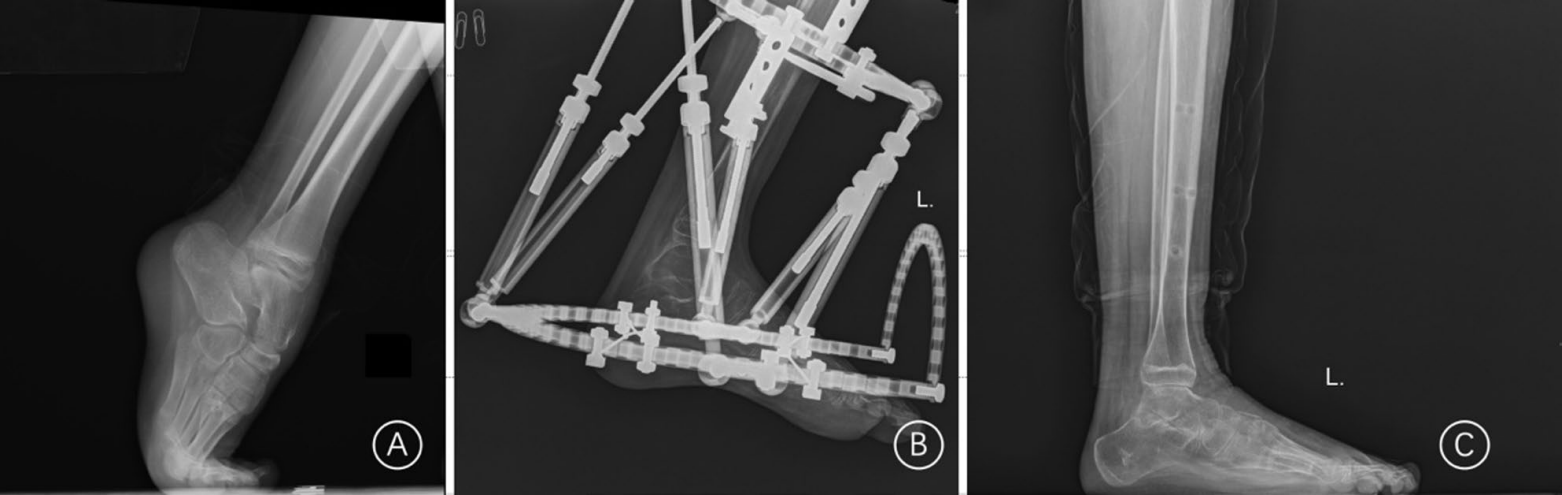
Typical case 5, rigid equinus deformity related to Fibro-Adipose Vascular Anomaly. **A** preoperative lateral X-ray films. **B** postoperative lateral X-ray films. **C** lateral X-ray films after fixator removal

Case 5 received local sclerotherapy for a left popliteal fossa hemangioma at age 1 year. The lesion recurred at age 4 years and was treated with surgical resection. Three months after resection, limited ankle dorsiflexion was noted. The hemangioma recurred again at age 7 years and was resected, after which the equinus deformity worsened. External fixation was performed at age 10–15 years. Preoperative and postoperative clinical photographs and radiographs of Case 5 are presented in Figs. 2 and 3.

The first follow-up assessment revealed the following: significant improvements were noted in the peak ankle dorsiflexion angle from preoperative − 43.4 ± 10.4 degrees (range − 57° to -32°) to follow-up 4 ± 6.5 degrees (range − 5° to 10°); however, the peak ankle plantarflexion angle did not show a significant difference, with preoperative values at 48.2 ± 10.1 degrees (range 36° to 62°) and follow-up at 37 ± 9.7 degrees (range 25° to 50°). Preoperative TTA was 145.0 ± 18.3 degrees (range 118° to 167°), and postoperative TTA was significantly improved to 106.8 ± 3.0 degrees (range 104° to 111°). There was also a significant increase in the average AOFAS score from 63.6 ± 4.6 (range 57 to 73) to postoperative 75.8 ± 8.0 (range 68 to 84), while the preoperative weight-bearing VAS score averaged 2 ± 1.2 (range 1 to 4), compared to the postoperative average of 0.8 ± 0.8 (range 0 to 2), with no significant difference observed (Table 2). Regarding complications, there were no severe neurovascular issues or infections. One patient experienced pain and discomfort at the pin sites, which was considered possibly related to local nerve damage or inflammatory response, warranting physiotherapy. Three patients reported difficulty in squatting during follow-up, and three patients reported varying degrees of pain while walking post-surgery. The patient satisfaction rate was 80%.

**Table 2:** Pre-operation and post-operation evaluation data

TTA 1	Preop.	Postop.	Z	*P*
	145.0 ± 18.3	106.8 ± 3.0	-2.203	0.043*
Peak ankle dorsiflexion angle 1	-43.4 ± 10.4	4 ± 6.5	-2.203	0.043*
Peak ankle plantarflexion angle 1	48.2 ± 10.1	37 ± 9.7	-1.841	0.066
AOFAS Score 1	63.6 ± 4.6	75.8 ± 8.0	-2.203	0.043*
VAS 1	2 ± 1.2	0.8 ± 0.8	-1.473	0.141
AOFAS Score 2	63.6 ± 4.6	77.2 ± 12.1	-2.023	0.043*
VAS 2	2 ± 1.2	1.4 ± 0.5	-1.134	0.257

*Abbreviations*: *1* first follow-up, *2* second follow-up, *AOFAS* American Orthopaedic Foot & Ankle Society ankle and hindfoot scale, *TTA* tibiotalar angle, *VAS* Visual Analog Scale

*P < 0.05

The second follow-up assessment revealed the following: preoperative AOFAS scores averaged 63.6 ± 4.6 (range, 57 to 73), while postoperative scores averaged 77.2 ± 12.1 (range, 61 to 93), with a statistically significant difference observed (*P* < 0.05). Preoperative weight-bearing VAS scores averaged 2 ± 1.2 (range, 1 to 4), compared to a postoperative average of 1.4 ± 0.5 (range, 0 to 2), with no statistically significant difference (*P* > 0.05) (Table 2).

Case 1 exhibited heel contact while standing but not during ambulation, with sharp pain in the forefoot after prolonged walking that resolved with rest. Compared to the post-frame removal status, the patient self-reported mild recurrence. The affected calf remained larger than the contralateral side, as the patient did not undergo concurrent hemangioma resection. The reason for only moderate satisfaction was attributed to the mild recurrence.

Case 2 demonstrated inability to achieve heel contact during ambulation, difficulty squatting, and sharp pain after walking 2,000 meters. Compared to the post-frame removal status, the patient self-reported severe recurrence, with a condition similar to the preoperative state. Postoperatively, the affected calf was noticeably thinner than the contralateral side. The change from moderate satisfaction to dissatisfaction was attributed to the severe recurrence.

Case 3 experienced sharp pain after walking 1,000 meters, which resolved with rest. Compared to the post-frame removal status, the patient self-reported mild recurrence. Postoperatively, the affected calf was noticeably thinner than the contralateral side. The reason for only moderate satisfaction was a discrepancy between the postoperative appearance and preoperative expectations.

Case 4 reported no specific discomfort or recurrence. Postoperatively, the affected calf was noticeably thinner than the contralateral side. The reason for only moderate satisfaction was weakness in the affected limb.

Case 5 experienced sharp pain during sprinting, which resolved with rest, with no recurrence. Postoperatively, the affected calf was noticeably thinner than the contralateral side. Patient satisfaction increased compared to the early follow-up period; the reason for only moderate satisfaction was that the affected foot had become smaller, requiring a shoe one size smaller than previously worn.

The mid-term patient satisfaction rate was 80%, with two patients experiencing mild recurrence and one patient experiencing severe recurrence.

## Discussion

In this study, gradual correction of rigid equinus deformity associated with FAVA using external fixation achieved significant improvement in ankle dorsiflexion, tibiotalar angle, and AOFAS scores at the first follow-up. At the second follow-up, the mean AOFAS score remained stable, suggesting that the acquired functional benefits were well-maintained over time. However, the efficacy of pain relief was limited, as weight-bearing VAS scores demonstrated no statistically significant reduction from the preoperative baseline at either follow-up interval. Recurrence of equinus deformity occurred in three patients, and patient satisfaction rate was 80%.

A decade ago, patients with FAVA were frequently misdiagnosed as having hemangiomas [3]. In 1937, Josefsson [10] first described a case of equinus deformity resulting from a calf muscle hemangioma. Over the years, without garnering significant attention, only sporadic case reports have highlighted intramuscular cavernous hemangiomas causing equinus deformity [1, 2, 6–8, 10–12]. In 2014, Alomari [3] introduced the concept of FAVA, distinguishing it from other congenital vascular malformations and detailed its clinical, radiological, and histological characteristics for the first time. FAVA commonly occurs in children and adolescents, often accompanied by muscle contracture and joint movement restriction. Besides venous malformations, a key feature of FAVA is fibro-adipose infiltration in skeletal muscles, leading to contractures and joint activity limit. Clinically and radiologically, FAVA can be categorized into three stages: pain, contracture, and deformity [4]. In 2018, FAVA was included in the ISSVA classification under unclassified vascular anomalies.

FAVA does not often affect the upper limbs and primarily occurs in the lower limbs [13], frequently within the gastrocnemius muscle, involving up to 44% of cases, with 40% affecting the soleus muscle [5, 14]. Alomari [3] reported that among 16 cases, 7 had calf muscle involvement that led to limited dorsiflexion of the ankle. In our study, we also noted a tendency for equinus deformity to worsen following previous FAVA resection. There has been little research on surgical treatment for FAVA-related deformities. In this study, external fixation was chosen based on clinical experience, as it offers greater comfort and reliability. Wang [14] reported five cases with third-stage involvement of ankle mobility, suggesting individualized treatment, including Achilles tendon lengthening and stretching exercises. Xie Chong [4] reported eight patients, four of whom had impaired ankle joint activity, aged 9–11 years, where one had previously undergone Achilles tendon lengthening without significant relief of pain and contracture. Xie Chong performed ankle arthrolysis and rehabilitation training for deformity correction; one received rehabilitation training, and two did not receive special treatment for limited ankle joint activity.

There is a scarcity of literature specifically discussing the external fixator treatment for this type of deformity. For the treatment of late-stage stiff deformities, there was only one study with 12 patients [15], where a patient with preoperative − 53 degrees equinus deformity was treated with external fixation combined with Hoke surgery, leaving a residual 17-degree deformity [7]. This study is pioneering in addressing external fixation for rigid equinus deformity related to FAVA, typically considered the end stage in FAVA progression. While various treatments exist, non-surgical methods like stretching or serial casting have limited efficacy for such rigid deformities [16], making surgical treatment the primary modality. Compared to surgeries involving extensive soft tissue release, external fixation is safer, entails fewer complications, and allows for more comprehensive correction [17, 18]. Commonly, a combination of procedures like Achilles tendon lengthening and gastrocnemius fasciotomy is employed to enhance outcomes [19]. However, Achilles tendon lengthening alone may lead to recurrence or require extensive lengthening, potentially causing complications like calf muscle weakness, leading to challenging conditions such as calcaneal gait [20, 21]. Both the Ilizarov apparatus and the Taylor Spatial Frame were used in this study. There is no essential difference between the two; comparatively, the Taylor Spatial Frame is more convenient to use and adjust.

At the first follow-up visit, there was a notable improvement in equinus deformity, and the AOFAS scores significantly increased from the preoperative scores. However, the improvement in pain, as measured by the VAS score, was not pronounced. Postoperative residual pain has been noted in other FAVA studies [14], with one report indicating that 80% of patients with third-stage ankle joint involvement experienced postoperative pain. Another study reported that 60% of patients had activity-related pain after FAVA resection, and 37% required analgesics post-surgery [5]. In our research, three patients reported reduced pain, one had no significant change, and one experienced increased pain, with the VAS score rising from 0 preoperatively to 1 postoperatively. Upon further inquiry, the patient reported soreness in the ankle joint after prolonged walking, suggesting the need for rehabilitative therapy and gait correction.

Some studies advocate for prophylactic decompression of the tarsal tunnel [22], but we did not perform this procedure on patients with severe equinus deformity, and no cases of tarsal tunnel syndrome occurred. This might be due to the lack of extensive adhesions in the medial soft tissues of the ankle, leading to minimal interference with the ankle joint during external fixation.

Our study has several limitations. First, our study was retrospective with a small sample size due to the rarity of rigid equinus deformity associated with FAVA. Additional multicenter prospective studies are needed to validate our study’s results. Second, the follow-up period was insufficient for some patients. However, to our knowledge, this is the first study about external fixation for rigid equinus deformity related to FAVA.

## Conclusion

This retrospective study demonstrates that the gradual correction of rigid equinus deformity related to FAVA using external fixation is satisfactory and safe, with minimal complications involving blood vessels, nerves or soft tissue, offering a valuable addition to the limited body of research in this area.

## Data Availability

The datasets generated and/or analysed during the current study are not publicly available due to patient privacy concerns but are available from the corresponding author on reasonable request.
